# Navigating Everyday as Partners to Persons With Early Dementia: A Scoping Review

**DOI:** 10.1111/scs.70089

**Published:** 2025-08-05

**Authors:** Mille Vogelius Bøtchiær, Hanne Kaae Kristensen

**Affiliations:** ^1^ University of Southern Denmark Odense Denmark; ^2^ Health Sciences Research Centre UCL University College London UK; ^3^ Centre for Innovative Medical Technology, Department of Clinical Research University of Southern Denmark Odense Denmark

**Keywords:** caregiving partners, early‐onset dementia, informal caregivers, literature review, lived experience, unmet needs, young‐onset dementia

## Abstract

**Background:**

Caregivers often lack access to comprehensive information on young‐onset dementia (YOD), leaving them unprepared for their caregiving roles. Especially, spousal caregivers of people with (YOD) face challenges, balancing caregiving with work and personal responsibilities in everyday life. However, their specific needs remain underexplored.

**Objective:**

This scoping review aimed to identify and map the lived experiences and unmet needs of spousal caregivers of persons with YOD.

**Methods:**

A systematic literature search was conducted across several databases (Embase, Scopus, Academic Search Premier, APA PsycInfo, CINAHL Ultimate, and PubMed via EBSCOhost), including MEDLINE to identify studies on spousal caregivers of people with YOD. Articles were screened and selected based on inclusion criteria, and key data were extracted and categorised into themes that captured the lived experiences and unmet needs of this caregiver population.

**Findings:**

The review identified five overarching themes related to the needs of spousal caregivers of people with YOD: psychoeducational, social support, practical support, emotional and psychological support, and navigational support needs. Caregivers often lack access to comprehensive information on YOD, leaving them unprepared for their caregiving roles. Social isolation, financial burdens, and role conflicts were common experiences. Emotional distress, identity loss, and lack of recognition for caregiving efforts contributed to psychological strain. Moreover, caregivers encountered significant challenges in accessing and coordinating healthcare services, often having to advocate for appropriate support. The need for tailored services, respite care, financial and legal guidance, and professional counselling was strongly emphasised.

**Conclusion:**

This scoping review identified a wide range of unmet needs among spousal caregivers of persons with YOD, underscoring the need for psychoeducational, social, practical, emotional, and navigational support. The findings revealed gaps in current support systems across personal, social, and systemic levels, emphasising the complex and disruptive nature of the caregiving experience. Future interventions should prioritise accessible, comprehensive, and tailored support services to effectively address these unique caregiving challenges.

## 1 Introduction

Dementia is an umbrella term for conditions in which the brain's cognitive functions significantly deteriorate, impeding the individual's ability to maintain activities of daily living (ADL) [1]. Globally, it is estimated that nearly 9.9 million people develop dementia annually and this number is projected to reach 75 million by 2030, increasing to 132 million by 2050 [1]. While dementia is often associated with ageing, the increased prevalence of the condition also occurs in individuals under the age of 65, referred to as early‐onset dementia/young‐onset dementia (YOD) [2, 3].

A meta‐analysis from 2021 estimates the global prevalence of dementia conditions in individuals aged 30–64 to be 119 per 100,000 population, equivalent to 3.9 million people worldwide [4]. Furthermore, the incidence rate is estimated to be 370,000 new cases of YOD annually [4]. People with YOD face a unique set of challenges, including significant barriers to diagnosis [5]. On average, it takes 1.6 years longer from the onset of symptoms to receive a diagnosis compared to those over 65, known as late‐onset dementia (LOD), resulting in an extended period of suffering, frustration, and uncertainty for both the individual and their relatives [6, 7, 8, 9]. Young‐onset dementia comprises a heterogeneous range of dementia diagnoses, including Alzheimer's disease, frontotemporal dementia, vascular dementias, and secondary dementias due to alcohol disorder, traumatic brain injury, and infections. The presentation of young‐onset dementia is also varied, with early presenting symptoms often consisting of behaviour, language, and personality change, and executive dysfunction. Furthermore, people diagnosed with YOD often encounter significant challenges related to work and finances. Many of them were previously self‐sufficient, active in the workforce, and co‐responsible for finances, household, and family, leading to significant changes in their life situation for both themselves and their families [2, 10, 11].

The onset of dementia early in life thus influences both the individual's health and daily functioning, as well as putting informal caregivers' health and well‐being at risk. As cognitive functions decline due to the disease, people with YOD increasingly require support, and research shows that it is primarily the informal caregivers, e.g., spouses, who take on this supportive and compensatory role, rather than healthcare professionals [12]. At the same time, informal caregivers of people with YOD often exhibit symptoms of stress and depression [8, 13, 14].

This burden is especially evident in caregiving partners, who, as the disease progresses, bear a double burden of work and caregiving responsibilities [12]. According to a national Swedish study from 2021, spouses of people with YOD more frequently take on caregiving tasks than other informal caregivers, with less support from family and formal services [15]. This often results in negative consequences for the spouses' social lives, as well as their mental and physical health. These findings resonate with prior studies, which found that 33% of caregiving relatives of people with dementia had significant depression symptoms [15, 16, 17]. Additionally, there are follow‐on effects on the mental health of children and young people in families with early dementia, with children lacking support and tools to cope with the upheaval caused by the disease [18, 19, 20, 21, 22]. Research shows that both offspring and partners of people with YOD face uncertainty, disruption of family life, and role reversals, as the informal caregivers take on responsibilities previously managed by the person with YOD [20, 23, 24, 25].

People with YOD utilise significantly fewer services from the social and healthcare systems than those with LOD, as informal care is used in a ratio of 3:1 compared to formal care [12]. Furthermore, it takes an average of 9 years from the onset of symptoms to moving to institutional placement, e.g., care facilities, whereas this occurs after an average of 4 years for people with LOD [26]. Informal caregivers express insufficient support from their surroundings, and it can be difficult to integrate their support needs into the general offerings, as existing services often cater to individuals in later life stages [27]. Thus, a significant portion of the responsibility for people with YOD falls on their caregivers, which may help explain why caregivers show signs of burden and pressure. Consequently, informal caregivers of people with YOD experience a significantly higher caregiver burden than those caring for people with LOD [14]. Of the informal caregivers, spouses provide significantly more care than other caregivers, providing an average of 30 h of care per week, against an average of 10 h or less for other caregivers [15]. Thus, emphasising the increased burden on this particular population.

This scoping review seeks to contribute to understanding the unmet support needs among spousal caregivers by systematically examining and mapping the lived experiences and challenges these caregivers face. To further illuminate these experiences, and to consider how they are shaped by social norms, interactions, and broader cultural contexts rather than occurring in isolation, this review draws on Michael Bury's concept of biographical disruption, which will be further explored in the discussion. This comprehensive overview could help inform the usage of a more evidence‐based approach to what future services and support should include, in what context it should be provided, and how and when it should be offered. There are no studies focused on caregiving spouses or partners of individuals with YOD; hence, underpinning the need for this scoping review.

### 1.1 Aim and Research Question

This scoping review aims to systematically identify, analyse, and map existing literature to explore the lived experiences and unmet needs of spousal caregivers in their roles as primary informal caregivers for people diagnosed with YOD. Accordingly, this review aims to lay the groundwork for future interventions that support the well‐being of informal caregivers and people with YOD.

The research question was: “What are the unmet needs and lived experiences of spousal caregivers of persons diagnosed with YOD in nonclinical settings such as everyday living, home life, community, and workplace environments?”

## 2 Methods

### 2.1 Design

The purpose of a scoping review is to systematically map and synthesise evidence from multiple sources, providing a comprehensive overview of existing literature [28]. This scoping review aims to identify gaps in knowledge and inform social and healthcare practice and policy. The review follows the Joanna Briggs Institute (JBI) methodology for scoping reviews to ensure a rigorous and structured approach [29]. Although critical appraisal is not required in scoping reviews, it will be conducted in this study to ensure the included literature's trustworthiness [30].

### 2.2 Eligibility Criteria

#### 2.2.1 Participants

This scoping review considered studies that focused on informal caregivers of people with YOD. The primary focus was on spouses or partners engaged in day‐to‐day caregiving responsibilities. Studies that involved patients, healthcare professionals, and/or caregivers who were not spouses or partners, such as children or other relatives, were excluded if the results could not be distinguished.

#### 2.2.2 Concept

The central concept of this scoping review was to explore the lived experiences and unmet needs of spousal caregivers in their roles as primary informal caregivers for people with YOD. Studies primarily focusing on the patient, medical treatments, or pharmacological interventions without addressing the caregiver's perspective were excluded.

#### 2.2.3 Context

This scoping review concerned everyday living in municipality settings, with a primary interest in caregivers living with a spouse or partner with YOD in their home environment. Studies including caregivers who had recently experienced their partner moving into a care facility were also considered, as their experiences continued to provide valuable insight into the topic investigated. Only studies from countries with healthcare systems comparable to Denmark, particularly those offering municipality‐based health services and publicly available support for people with dementia and their caregivers, were considered. This criterion was chosen to enhance the transferability of findings to the Danish context. By being transparent about this selection, we aim to support readers in assessing the relevance and applicability of the findings within their settings. Studies conducted in hospitals were excluded.

#### 2.2.4 Types of Sources

All study designs were eligible for inclusion in the scoping review to ensure all relevant material related to the phenomenon of interest. Only studies published in English and the Scandinavian languages (Danish, Norwegian, and Swedish), between 2013 and 2023, were considered.

### 2.3 Search Strategy

A thorough literature search was conducted for published studies related to the lived experience and unmet needs of spousal caregivers of persons with YOD. Initially, the research question was analysed and divided into four aspects: dementia, next‐of‐kin, experience, and intervention. This was followed by a preliminary search in PubMed and CINAHL to identify relevant keywords and index terms for each aspect. To ensure accurate terminology, the identified keywords were analysed and looked up in the thesaurus in PubMed. These terms guide the full search strategy across all selected databases. A search protocol was conducted.

The systematic search was executed as a block search, utilising Boolean operators (AND/OR) to combine free text with the indexed terms, and was adapted as needed for each database. Search limitations were set to the year of publication. An example of a search string is available in Table 1.

**TABLE 1:** Example of search string.

Database	Search string	Hits	Limitations
PubMed	((((((“early onset dementia”) OR (“early onset alzheimer”)) OR (“young onset dementia”)) OR (“young onset alzheimer”)) AND ((((((((((((spouse) OR (spousal)) OR (husband)) OR (wife)) OR (married)) OR (partner)) OR (care‐partner)) OR (family)) OR (loved ones)) OR (next‐of‐kin)) OR (relatives)) OR (informal caregivers))) AND ((((((((quality of life) OR (psychosocial)) OR (lived experience)) OR (well‐being)) OR (emotion*)) OR (Coping)) OR (needs)))) AND (((((((support) OR (support‐system)) OR (program)) OR (intervention)) OR (training)) OR (skills)) OR (strategies))	366	Published 2013–2023

The databases searched included: Embase, Scopus, Academic Search Premier, APA PsycInfo, CINAHL Ultimate, and PubMed via EBSCOhost, including MEDLINE.

The search strategy was conducted and discussed in the research team in collaboration with an experienced librarian.

The full search strategy is provided in Appendix A.

### 2.4 Study Selection Process

**FIGURE 1:**
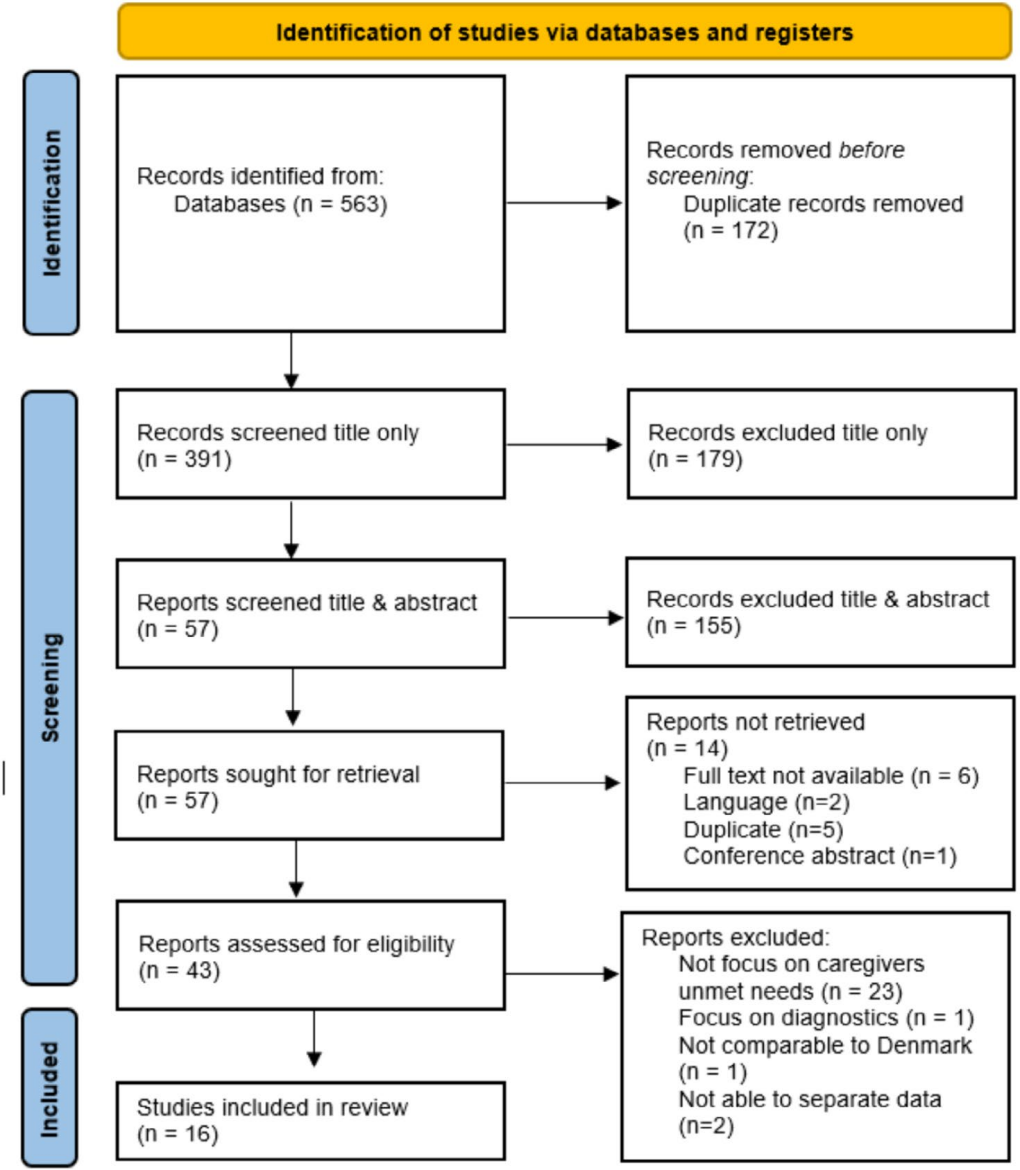
PRISMA flowchart.

After completing the search, a total of 563 studies were identified and uploaded into EndNote [31], where a total of 172 duplicates were removed. 391 studies were then screened for title only, to remove studies that did not meet the inclusion criteria and studies ineligible due to language criteria. The 212 remaining studies were uploaded to the JBI System for the Unified Management of the Assessment and Review of Information [32], where two reviewers assessed abstracts against the predefined inclusion criteria. This process rendered 57 studies, which were sought for retrieval. Of these, a total of 43 studies were retrieved and read in full text. The full‐text reading yielded 16 studies for inclusion in the scoping review. All ambiguity that arose regarding eligibility was discussed and resolved within the research team. The search process and reasons for exclusion are illustrated by a PRISMA flow diagram [33] and are available in Figure 1.

The initial search was conducted in mid‐2024 and re‐searched again in January 2025. No further studies were eligible for inclusion.

### 2.5 Critical Appraisal

Although not a requirement for scoping reviews, the included studies were assessed utilising two recognised appraisal tools: the Critical Appraisal Skills Programme (CASP) for Qualitative Research [34] and the JBI Checklist for Analytical Cross‐Sectional Studies [35]. The CASP tool facilitates the assessment of the qualitative studies included in the review, while the JBI checklist is used for the cross‐sectional study. The tools consist of 10 and 8 questions, respectively, that help evaluate the methodological trustworthiness of the studies before data extraction and chartering.

**FIGURE 2:**
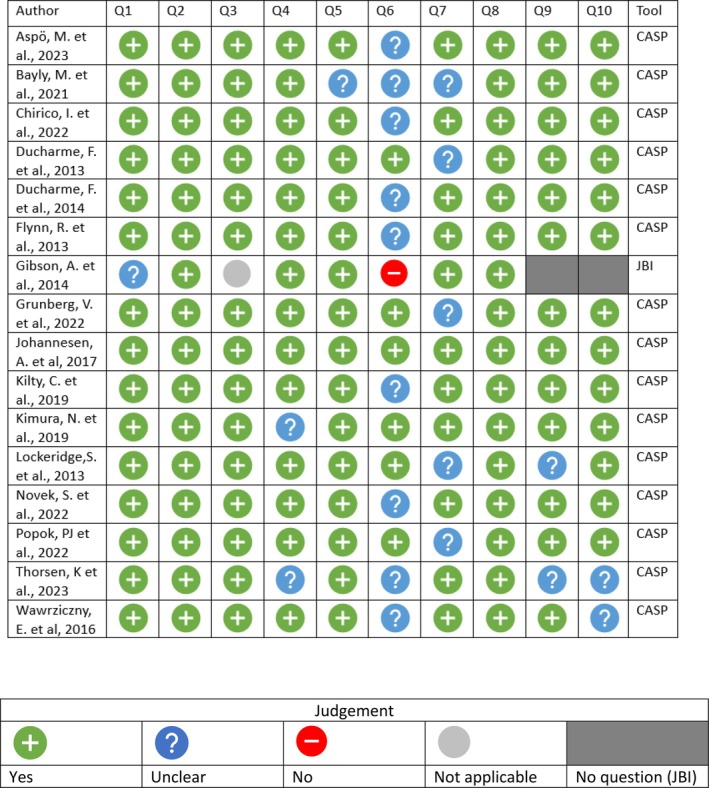
Critical appraisal CASP & JBI.

A visual illustration is available in Figure 2, and the full critical appraisal is available in Appendix B.

## 3 Extracting and Charting the Results

The data extraction process for this scoping review was initially conducted jointly by both researchers for the first five studies. The remaining 11 studies were extracted by a single researcher, after which all extracted data were reviewed together before analysis. A charting table was used in alignment with the JBI methodology for scoping reviews [29]. Key information extracted from each study included details about author(s), objective(s), country, study design, participant demographics, concept, and context. Findings related to the lived experiences, unmet needs, and strategies sought by spousal caregivers of persons with YOD were extracted in a separate charting form.

An inductive content analysis, following Elo and Kyngäs's framework [36], was conducted to systematically code and cluster the extracted data, facilitating an open‐ended exploration and identification of patterns within the reported narratives of the informal caregivers. This inductive approach is especially suited for less explored phenomena and enables a structured yet flexible process of coding and clustering the narratives, allowing for the identification of recurring patterns and themes within the caregivers' accounts. The research team employed an iterative and collaborative approach to facilitate the refinement of these themes, incorporating researcher triangulation to enhance the credibility and reliability of the analysis. By systematically reviewing and discussing interpretations, the team ensured that the findings were both relevant and reflective of the diverse experiences shared across the studies. This process led to the categorisation of findings into coherent, overarching themes, providing a comprehensive synthesis of the unmet needs of the caregivers.

### 3.1 Mapping of the Included Articles

A total of 16 studies were included in this scoping review originating from Canada (4), USA (3), Ireland (2), Norway (2), Sweden (1), Italy (1), United Kingdom (1), Brazil (1), and France (1). An extraction was conducted to summarise key information about each article, including the author, context, participants, type of dementia of the person with YOD, and relevant findings. These summaries are presented in Table 2. The 16 studies included data from a total of 373 participants, consisting of people with YOD, caregiving partners to people with YOD, other caregivers such as children or other relatives, and healthcare professionals. The number of informal caregivers totalled 316, of which 206 were female and 110 were male. Due to the nature of this scoping review, only data from caregiving partners to people with YOD were included in the data extraction and chartering, adding up to a total of 251 participants in this scoping review.

**TABLE 2:** Overview of included studies.

# ID, author, year of publication	Title	Country	Study design	Context living conditions (institution—non institution)	Number of participants	Gender of caregivers	Age of participants in years	Type of dementia	Relevant extractions
#1 [37]	Family Members' Experiences of Young‐Onset Dementia: Becoming Responsible Yet Feeling Powerless	Sweden	Qualitative approach with in‐depth interviews and thematic analysis	Nursing home	Fifteen relatives to 13 persons with YOD. 9 were partners, 3 were adult children, and 3 were siblings. 8 participants lived together with the person with YOD prior to the nursing home	Female (*n* = 9) Male (*n* = 6)	The relatives had an average of 52 years when a person with YOD was diagnosed	9 ad, 2 FTD, 2 WKS	The partners needed safe spaces for themselves and for the persons with YOD, personalised support, and guidance. The partners emphasised the need for aid in decision‐making. There was expressed a need for expert professionals
#2 [38]	Family carers' narratives of the financial consequences of young onset dementia	Canada/USA	Qualitative narrative study	N/A	8 relatives to persons with YOD. 5 were partners, 2 were adult children, and 1 was the niece of a person with YOD	Female (*n* = 7) Male (*n* = 1)	The relatives had a mean age of 52 at the time of the interview	5 ad, of which 1 also had aphasia, 1 with both LBD and PD, 1 PCA, 1 dementia	Discovered a major gap in financial support for YOD. Financial consequences started pre‐diagnosis and continued in ongoing life with YOD due to loss of employment or early retirement
#3 [39]	Family experience of young‐onset dementia: the perspectives of spouses and children	Italy	Qualitative approach with semi‐structured interviews	N/A	38 relatives of persons with YOD. 26 were spouses and 12 were adult children. All from different families	Female (*n* = 26) Male (*n* = 12)	The mean age of spouses was 65.1 years. The Adult children were on average 35.5 (±9.4) years old	ad 65.8%, FTD 23.7%, unknown dementia 10.5%	The spouses of individuals living with YOD had unmet needs related to information and counselling, post‐diagnostic support, emotional and social support, access to appropriate care services, and assistance with family dynamics
#4 [40]	The Unique Experience of Spouses in Early‐Onset Dementia	Canada	Qualitative approach with semi‐structured interviews	Resides at home	12 participants, all spouses to the person with YOD	Female (*n* = 8) Male (*n* = 4)	Mean age of 55 years	9 AD, of which 2 of these also had LBD and VD, respectively, 3 PD and MD	The unmet needs of spouses caring for individuals with YOD encompassed a lack of tailored support services, challenges in symptom management, a need for social connection and stigma reduction, difficulties in balancing multiple roles, planning for the future, and support during their identity transition
#5 [41]	Unmet support needs of early‐onset dementia family caregivers: a mixed‐design study	Canada	Mixed method design	Resides at home	32 participants. 25 of these were spouses to the persons with YOD, 3 were offspring and 2 were other (N/A)	Female (*n* = 24) Male (*n* = 8)	Mean age of 54.28 years	19 ad, 8 FTD, 5 other (N/A)	A significant number of caregivers to persons with YOD reported unmet support needs. Particularly in the areas of information on available help and financial resources, to reduce stress stemming from their caregiver role, and to receive the right help at the right time. Furthermore, they wished to have meaningful activities and connections for the persons with YOD
#6 [42]	Early‐onset dementia: the impact on family care‐givers	Ireland	Qualitative approach with semi‐structured interviews	Living at home	7 participants. 5 of these were spouses to the persons with YOD, 1 was an adult child, and 1 was other relations	Female (*n* = 2) Male (*n* = 5)	2 of the participants were in the age group of 31–55 years, the 5 remaining five participants were between 56 and 65 years	N/A	The caregivers needed specialised services in the community to aid in the changes caused by YOD – these should have involved the family as a unit. These services should be multi‐disciplinary and tailored to YOD, including guidance and information about YOD, relationship changes, psychosocial support, and financial aid
#7 [43]	Exploring the service and support needs of families with early‐onset alzheimer's disease	USA	Cross‐sectional	N/A	81 participants. Of these, 56 were spouses to the persons with YOD, 17 were adult children, and 8 had other relations to the person with YOD	Female (*n* = 62) Male (*n* = 19)	N/A	Only AD	Overall service utilisation is low for families affected by YOD. Almost half of the caregivers did not feel like the service providers understood their needs, and the majority did not feel like the general population understood their needs
#8 [44]	Psychosocial treatment preferences of persons living with young‐onset dementia and their partners	USA	Qualitative approach with dyadic interviews	Living together	23 couples, which means there were 46 participants in total (23 persons living with YOD and 23 partners)	Female (*n* = 13) Male (*n* = 10)	Mean age 60.52 years	12 ad, 8 FTD, 2 PSP, 1 unknown	The partners articulated a strong need for tailored psychosocial interventions right after diagnosis that addressed their unique experiences and challenges as a couple, emphasising the importance of flexibility, communication skills, and ongoing support
#9 [45]	Experiences and needs of spouses of persons with young‐onset frontotemporal lobe dementia during the progression of the disease	Norway	Qualitative design	N/A	16 participants. 15 were spouses, 1 was a male cohabitant	Female (*n* = 9) Male (*n* = 7)	Mean age 59.56 years	Only FTD	The spouses of persons with YO‐FTD experienced a personality change in their partner at symptom onset and had a difficult time getting a diagnosis, and were often met by disbelief from professionals. As the disease progressed, the spouses became increasingly burdened, isolated, mentally exhausted, and lonely. They needed help to get relief from the care burden and wished for coordinators to aid in, e.g., decision‐making and administrative tasks
#10 [46]	Caring for People With Young Onset Dementia: An Interpretative Phenomenological Analysis of Family Caregivers' Experiences	Ireland	Qualitative design	N/A	6 participants. 3 spouses, 1 sibling, 1 adult child, and 1 unknown relation	Female (*n* = 3) Male (*n* = 3)	Mean age 55 years	N/A	The carers felt difficulty obtaining a diagnosis and were strained from the long process. After the diagnosis came a clarity followed by grief, distress, and overwhelm. The carers expressed a need for tailored support to both the person with YOD and the caregivers, to reduce isolation. The carers expressed a need for help to navigate
#11 [13]	Psychosocial impact of early onset dementia among caregivers	Brazil	Qualitative design	N/A	9 participants of these, 4 were spouses, 1 was a partner, 3 were adult children, and 1 was a sibling	Female (*n* = 7) Male (*n* = 2)	Mean age 51.22 years	Only AD	Caregivers of persons with YOD often assumed the role prematurely, having to balance caregiving with other responsibilities. Their unmet needs span emotional, financial, informational, and social aspects, underscoring the importance of tailored support systems to assist them in these challenges
#12 [47]	The experience of caring for a partner with young onset dementia: How younger carers cope	United Kingdom	Qualitative design	Living together	6 participants, all spouses of a person with YOD. 4 were currently caregiving at home, while 2 were widowed, as their spouse with YOD had passed away by the time of the interview	Female (*n* = 3) Male (*n* = 3)	Mean age 63 years	N/A	The spouses faced stigma and sought different coping strategies, such as denial, to manage with their partners YOD. Unfortunately, these sometimes led to unwanted consequences later, with a lack of trust. The partners felt isolated and lost control. They had to mourn their partners while taking care of them
#13 [48]	Conceptualising access to community‐based supports from the perspectives of people living with young onset dementia, family members and providers	Canada	Qualitative design	Own home	20 participants. 6 persons with YOD and 14 family members; 11 spouses, 2 adult children, and 1 niece. 16 professionals who work with the YOD population	Female (*n* = 7) Male (*n* = 7)	Family members' age range is 20–76 years	Person with YOD: 4 ad, 1 LBD, 1 unknown Relatives: 10 ad, 1 LBD, 1 FTD, 2 unknown	The spouses of persons with YOD underscored a fragmented and uncoordinated system, where the relevant services were difficult to find and often not tailored to persons with YOD. They found restrictions and were unable to get the support they needed to cope with the YOD, which added further to a stressful and overwhelming situation
#14 [49]	One Diagnosis, Two Perspectives: Lived Experiences of Persons With Young‐Onset Dementia and Their Care‐Partners	USA	Qualitative design	Co‐habitating	29 participants. 17 Care partners. 12 persons with YOD	Female (*n* = 9) Male (*n* = 8)	Mean age of care partners: 61.47 years	Persons with YOD: 3 AAD, 2 AD, 2 PCA, 2 PSP, 2 PPA, 1 FTD Relatives: N/A	The spouses faced many stressors associated with YOD. They needed help from both professionals and their network to navigate, cope, and accept the changes. They emphasised the need for knowledge about YOD in both personal and professional relations. Having time for self‐care, activities, and social support was an efficient coping strategy
#15 [50]	How gender matters in demanding caring for a spouse with young‐onset dementia. A narrative study	Norway	Narrative qualitative approach	Living together. 5 spouses had moved permanently into nursing homes at the time of the interviews. One had passed away	16 participants. These comprised 10 wives, 5 husbands, and 1 male cohabitant	Female (*n* = 10) Male (*n* = 6)	The mean age of the women was 58.3 years, and 61.7 years for the men	Only FTD	The spouses needed support and relief in caring for their partner with YOD. All spouses felt distress, but male partners were quicker to seek relief and aid from public care than their families. The female caregivers struggled longer and felt a loss of identity while caring for their partner for longer periods. Especially a need for support to maintain the sense of self/self‐care
#16 [51]	From ‘needing to know’ to ‘needing not to know more’: an interpretative phenomenological analysis of couples' experiences with early‐onset Alzheimer's disease	France	Qualitative approach with dyadic interviews	Living together	32 participants, 16 couples	Female (*n* = 7) Male (*n* = 9)	Mean age 57.4 years at time of interview	Only AD	The care partners went from ‘needing to know’ at symptom onset, to ‘not needing to know more’ after the diagnosis was finally given. They took on an avoidant strategy to minimise pain and suffering; however, it seemed to just move to the background. They needed help with effective coping strategies, disease information, and emotional support

Abbreviations: AAD, Atypical Alzheimer's disease; AD, Alzheimer's disease; FTD, Frontotemporal dementia; LBD, Lewy Body dementia; MD, Mixed dementia; PCA, Posterior cortical atrophy; PD, Pick's disease; PPA, Primary progressive aphasia; PSP, Progressive supranuclear palsy; WKS, Wernicke‐Korsakoff's syndrome.

### 3.2 Map of Findings

This scoping review sought to map and synthesise the unmet needs and lived experiences of spousal caregivers of persons with YOD. Through the data extraction and analysis, five overarching themes were identified: psychoeducational needs, social support needs, practical support needs, emotional and psychological support needs, and navigational and societal needs. These themes encompass the multifaceted challenges and provide valuable insight into the caregiving experience and the complexities that spousal caregivers face. A single study investigated the gender differences in caregiving approaches among spousal caregivers of people with YOD. These findings are presented in Table 8.

A recurrent element within the narratives was the implicit desire of the spousal caregivers to maintain their everyday lives as they were pre‐diagnosis. Many of the unmet needs occurred from an underlying struggle to preserve normalcy while simultaneously facing the disruption that YOD causes on family dynamics, routines, and future plans. Thus, it became evident that these needs and challenges did not occur in isolation. However, they emerged from the disruptions in everyday life due to YOD.

Each of the overarching themes will be delineated and presented in the following section. To ensure an accessible overview, the findings from the scoping review will be presented in both tabular and narrative formats.

#### 3.2.1 Psychoeducational Needs

Psychoeducational needs refer to the spousal caregivers' need to acquire knowledge, skills, and coping strategies to ensure the best grounds to take on the caregiving role. This encompassed early intervention programmes, information about YOD, training in relevant caregiving skills, and support to develop resilience. Table 3 below outlines the specific subthemes and findings related to psychoeducational needs.

**TABLE 3:** Psychoeducation and skills findings.

Subtheme	Findings	Study ID #
Information and knowledge about YOD	Needs for accurate and timely information on the diagnosis, progression, and impact of YOD	1, 3, 4, 5, 6, 8, 9, 10, 11, 12, 13, 14, 15, 16
Training in caregiver skills	Developing practical caregiving skills for communication, managing behaviours, and evolving care needs of people with YOD	1, 3, 4, 5, 6, 8, 9, 10, 11, 12, 13, 14, 15, 16
Learning coping strategies	Wishes for strategies to handle emotional and practical caregiving demands, including stress management and emotional resilience	1, 4, 6, 8, 9, 10, 11, 12, 13, 14, 15, 16
Early intervention and guidance	Emphasis on early‐stage support and programs for caregivers to adjust and prepare for the future	1, 3, 4, 5, 8, 9, 10, 12, 13, 14, 15, 16
Developing resilience and self‐care practices	Learning self‐care routines and building resilience to manage caregiver stress and improve physical and emotional health	1, 2, 4, 5, 6, 8, 9, 10, 11, 12, 13, 14, 15, 16

#### 3.2.2 Social Support Needs

The social and relational findings encompass the spousal caregivers' need for connection, understanding, and emotional support. The informal caregivers experienced isolation and a shift in their personal and relational roles, enhancing the importance of tailored support groups to YOD. The key findings of the social support needs are presented in Table 4 below.

**TABLE 4:** Social and relational findings.

Subtheme	Findings	Study ID #
Social isolation and disconnection	Caregivers often experience social isolation due to caregiving demands and a perceived lack of understanding from social networks	1, 2, 3, 4, 5, 6, 8, 9, 10, 11, 12, 13, 14, 15, 16
Need for social connections and peer support	Wishes for social interactions and peer support from friends and family	1, 2, 3, 4, 5, 6, 7, 8, 9, 10, 11, 12, 13, 14, 15, 16
Support groups tailored to YOD	Caregivers seek support groups, specifically for YOD, to share experiences, receive emotional support, and establish social connections	1, 2, 3, 4, 6, 8, 9, 10, 11, 12, 13, 14, 15, 16
Role transition and identity loss	Loss of personal identity and redefining self as a caregiver, with the sense of being overwhelmed and taking on all family roles and responsibilities	1, 3, 4, 6, 8, 9, 10, 11, 12, 13, 14, 15, 16
Loss of intimacy and relationship strain	The spousal relationship shifts from partnership to caregiver/care recipient, resulting in loss of intimacy and emotional connection	1, 2, 3, 4, 6, 8, 9, 10, 11, 12, 14, 15, 16

#### 3.2.3 Practical Support Needs

Practical support needs refer to the challenges spousal caregivers face in managing daily living tasks alongside their caregiving responsibilities. This theme included balancing caregiving with work and personal life, addressing financial strain, and accessing respite care and tailored support services. Table 5 below highlights the subthemes and findings associated with practical support needs.

**TABLE 5:** Practical and daily living findings.

Subtheme	Findings	Study ID #
Balancing work‐life and caregiving	Difficulty in balancing caregiving responsibilities with employment and other personal responsibilities	1, 2, 3, 4, 5, 6, 8, 9, 10, 11, 12, 13, 14, 15, 16
Need for respite care and breaks	Needs for temporary relief from caregiving through respite services, day programs, and other support	1, 2, 4, 5, 6, 8, 9, 10, 12, 13, 14, 15, 16
Financial strain and need for assistance	Financial pressure due to loss of income, increased caregiving costs, and a need for financial assistance and support	1, 2, 4, 5, 6, 8, 9, 10, 11, 12, 13, 14, 15, 16
Tailored support services for YOD	Needs for specialised and age‐appropriate services that address the unique needs of people with YOD and their caregivers	1, 3, 4, 5, 6, 8, 9, 10, 12, 13, 14, 15, 16
Constant vigilance and exhaustion	Physical and mental exhaustion from continuous caregiving and the sense of “constant surveillance” over the person with YOD	1, 2, 3, 4, 5, 6, 8, 9, 10, 11, 12, 13, 14, 15, 16
Neglect of Self‐Care	Many caregivers reported neglect of their health and well‐being, focusing entirely on their spouse's care and losing the time and energy for self‐care activities	1, 2, 3, 4, 5, 6, 9, 10, 11, 12, 13, 14, 15, 16

#### 3.2.4 Emotional and Psychological Support Needs

The emotional and psychological support needs reflected the mental and emotional challenges faced by the spousal caregivers, including stress, grief, and guilt. This theme underscores the need for caregivers to feel valued and recognised in their roles. Moreover, the importance of professional support and safe spaces to process the emotional and psychological burdens of caregiving is highlighted. Table 6 below presents the key findings and subthemes.

**TABLE 6:** Emotional and psychological findings.

Subtheme	Findings	Study ID #
Emotional burden and mental health challenges	High levels of stress, anxiety, grief, guilt, and emotional exhaustion due to caregiving duties and role changes	1, 2, 3, 4, 5, 6, 8, 9, 10, 11, 12, 13, 14, 15, 16
Grieving the altered future	Mourning the loss of plans for the future, lifestyle, and expected life trajectory prior to YOD diagnosis	1, 3, 4, 6, 8, 9, 10, 11, 12, 13, 14, 15, 16
Safe spaces for caregivers to process emotions	Caregivers need safe spaces to share and process their experiences and emotions, often requiring support for grief and acceptance	1, 3, 4, 5, 6, 8, 9, 10, 11, 12, 13, 14, 15, 16
Need for recognition and validation	Caregivers sought acknowledgment and validation for their caregiving efforts and emotional challenges	1, 3, 4, 5, 6, 8, 9, 10, 11, 12, 13, 14, 15, 16
Professional counselling and emotional support	Seeking access to professional counselling and support services to help process the emotions and challenges of caregiving	1, 4, 6, 8, 9, 10, 11, 12, 13, 14, 15, 16

#### 3.2.5 Navigational and Societal Needs

Navigational and societal needs highlighted the complexities that spousal caregivers face in accessing and coordinating care for their partners with YOD. These challenges included navigating complex healthcare and social systems, managing legal and financial matters, and the need for broader societal awareness and understanding of YOD to reduce stigma. Findings and subthemes related to these challenges are presented in Table 7 below.

**TABLE 7:** Navigational and societal findings.

Subtheme	Findings	Study ID #
Pre‐diagnosis difficulties	Uncertainty, distress, and a need for knowledge as to why a partner was undergoing changes. Need for accurate diagnosis	3, 4, 6, 9, 10, 12, 16
Challenges in accessing healthcare services	Difficulty finding appropriate care and navigating complex healthcare systems to meet the needs of people with YOD	1, 2, 3, 4, 5, 6, 8, 9, 10, 12, 13, 14, 15, 16
Need for service coordination and guidance	Caregivers needed support to navigate and coordinate healthcare, social services, and other support systems, as well as guidance for making difficult choices such as transitioning to nursing homes	1, 2, 3, 4, 5, 6, 8, 9, 10, 12, 13, 14, 15, 16
Feeling Overwhelmed by Bureaucratic Processes	Frustration with navigating complex systems, fragmented services, and feeling burdened by the “navigation work”	1, 2, 4, 5, 6, 8, 9, 10, 12, 13, 14, 15, 16
Financial and legal guidance	Needs for assistance with financial planning, accessing benefits, and managing legal aspects related to caregiving	1, 2, 3, 4, 5, 6, 8, 9, 10, 11, 12, 13, 14, 15, 16
Awareness and understanding in society	Enhanced societal understanding of YOD to reduce stigma, promote awareness, and improve social support networks for caregivers	3, 4, 5, 6, 7, 8, 9, 10, 11, 12, 13, 14, 15, 16

#### 3.2.6 Gendered Differences

A single study, #15 [50], provided unique insights into the gendered experiences of caregiving for partners with YOD. Women faced higher emotional burdens, career disruptions, and delays in seeking external support, whereas the men tended to seek help sooner, maintain a work‐life balance, and approach caregiving more practically. Table 8 below summarises these key findings.

**TABLE 8:** Gender‐specific findings.

Key findings	Female spousal caregivers	Male spousal caregivers
Emotional Burden	Higher levels of stress, exhaustion, guilt, and feeling isolated	Experience stress but less emotional burden; seek support proactively
Impact on Work	Greater disruption to careers; many reduced work or left their jobs	Maintain work‐life balance; less impact on employment
Seeking Support	Delay seeking help, leading to prolonged caregiving at home	Seek public and personal support sooner, especially for care needs
Role and Identity	Strong identity as a nurturer; caregiving affects self‐identity and causes emotional distress	Practical approach to caregiving; identity was less affected by caregiving role changes

## 4 Discussion

The findings of this scoping review highlight a substantial need for structured support tailored specifically to spousal caregivers of people with YOD across several domains, including informational, emotional, and practical support. It also shows that the needs of these caregivers span across personal, social, and systemic levels, emphasising the complexity and depth of the caregiving experience. The results furthermore reflect the lack of comprehensive, accessible support and clear pathways for spousal caregivers, who frequently face fragmented and insufficient social and health services, which add to their burden and often leave them feeling distressed and unsupported.

### 4.1 Disruption of Everyday Living

Many of the unmet needs identified in this review stem from the underlying struggle to preserve normalcy while grappling with the multifaceted and profound changes YOD causes in family dynamics and daily living. To better understand the findings of the review, sociologist Michael Bury's concept of biographical disruption provides a useful framework for examining the lived experiences of spousal caregivers of persons with YOD. Although originally applied to chronic illness, biographical disruption finds relevance in highlighting the impact of YOD on informal caregivers' lives. Bury describes three stages of disruption: (1) the breakdown of everyday life structures, (2) the rethinking of identity and future plans, and (3) the mobilisation of resources to adapt to these changes [52]. Each phase reflects the unmet needs and challenges faced by spousal caregivers, as identified in this review.

#### 4.1.1 Stage 1: “What is Going on?”

In the first stage of biographical disruption, a subtle but destabilising breakdown of everyday routines unfolds, as the caregivers begin to notice changes in their partner's behaviour. Often, spousal caregivers are the first to realise that something is wrong, but the lack of timely diagnosis and clarity about the condition leaves them in a state of uncertainty. Many caregivers report feeling lost, distressed, and overwhelmed as they attempt to make sense of what is happening, without clear diagnoses or support from healthcare systems. Thus, they must navigate the breakdown of normalcy in everyday life while searching for answers. This phase often ends with the diagnosis of YOD, but the uncertainty and disruption continue into the next stage.

#### 4.1.2 Stage 2: Rethinking Identity and Future

Once a formal diagnosis is received, the informal caregivers enter the second stage of biographical disruption, where they must fundamentally rethink their roles, identity, and future. The diagnosis does not resolve all uncertainties, as caregivers face a lack of adequate information, support, and guidance. As YOD progresses, spousal dynamics shift, as the person with YOD becomes increasingly dependent on their partner, who often takes on the informal caregiving role prematurely, without proper knowledge, support, or guidance. Thus, spouses who once were partners in reciprocal relationships undergo a shift from partnership to caregiver/care recipient, resulting in the loss of intimacy and emotional connection. Adapting to their new role as a caregiver, while undergoing the emotional toll of losing their partner as the person they once knew, exacerbates a sense of loss, impacting their mental health and well‐being. Moreover, practical challenges, such as balancing caregiving responsibilities with work and personal life, securing financial and legal assistance, and accessing respite care, further amplify the burden of care. Thus, the onset of YOD disrupts not only everyday structures but also relationships, and material and practical affairs, leaving the caregiving partners to fundamentally rethink identity and future.

#### 4.1.3 Stage 3: Mobilisation of Resources

In the final stage of biographical disruption, the informal caregivers attempt to mobilise resources and restore a sense of order and normalcy to their disrupted lives. However, the findings of this review indicate that many face a lack of structured, age‐appropriate support tailored to the unique needs of YOD. This leaves the spousal caregivers struggling to find appropriate services, coordinate care, and navigate complex healthcare systems and social services. Furthermore, a disruption of their social lives takes place, as the spousal caregivers find it difficult to attend and maintain normal social activities due to caregiving demands and a perceived lack of understanding from social networks. The cumulative burden results in a heightened risk of mental health issues, social isolation, neglect of self‐care, and an overall decrease in quality of life (1–6, 8–16). While the mobilisation of resources theoretically offers a way to restore order, it remains potentially unattainable for many caregivers due to fragmented services and a lack of comprehensive support specifically tailored to the challenges of YOD. Thus, it becomes evident that spousal caregivers undergo a long‐lasting disruption of their lives and identity, which necessitates both emotional, social, and practical adaptations. Each stage of disruption presents new challenges, where spousal caregivers face complex support needs. These findings underscore the necessity of providing tailored support that helps informal caregivers navigate the fundamental upheaval of their lives.

### 4.2 Informal Caregiving in Society

The strain experienced by spousal caregivers is of great relevance when considering the broader role of informal caregiving in society. The World Health Organisation (WHO) emphasises that informal caregivers are essential to the healthcare systems and society at large in European countries, not only for the well‐being of the care recipients but also for economic sustainability [53]. The economic value of time spent on informal care in Europe accounts for approximately 3.6% of GDP (Gross Domestic Product), exceeding public expenditure on formal long‐term care services in most European Union countries [53, 54]. This highlights a significant economic incentive to support informal caregivers and ensure they are thriving and able to continue their caregiving role, without sacrificing their health and well‐being, as it is both a societal benefit and a cost‐effective approach to care.

However, the findings of this review suggest that current social and healthcare systems fail to support these informal caregivers effectively. Often, they must sacrifice personal goals, social lives, and professional ambitions to accommodate the increasing needs of their partner with YOD, whilst feeling stressed, overwhelmed, and exhausted (1–16). A key aspect of informal caregiving for people with YOD is the need for constant vigilance, referring to the sustained attentiveness required to monitor the safety, behaviour, and evolving needs of the person with YOD, often resulting in mental strain and physical exhaustion. A Dutch study by Bakker et al. [12] underscores this issue, finding that supervision and surveillance constituted the largest part of informal care for persons with YOD. Thus, balancing employment and caregiving duties is exceptionally challenging. This aligns with WHO findings indicating that 1 in 10 adult caregivers in the European Union has been forced to reduce their working hours or quit their jobs entirely to provide care [55]. This underlines the importance of providing tailored support to enable informal caregivers to balance their responsibilities without sacrificing their professional and personal lives.

#### 4.2.1 Gendered Imbalance

Gendered differences in informal caregiving have been acknowledged, but as this perspective is relatively new, only a single study directly addressed it. However, this does not diminish its relevance, as 65.2% of the informal caregivers in this scoping review were female. This aligns with data from WHO Europe, which finds that women are more likely to be unpaid informal caregivers, with the gender disparity being most significant between ages 50–64 [53, 55]. While providing informal care for their partners with YOD, female caregivers often endure greater physical and emotional strain over longer periods compared to their male counterparts, leading to poorer self‐rated health and a higher emotional toll, and their needs are more likely to be overlooked [50, 56]. These findings suggest that tailored interventions must not only address the life stage and challenges of YOD but also consider the gender‐specific differences in caregiving experiences and needs, which is also emphasised by WHO Europe [57].

### 4.3 Tailored Support

A crucial finding of this review is the need for tailored support that addresses the unique challenges faced by spousal caregivers to people with YOD, such as the early onset of symptoms and the resulting disruption to expected life roles, relationships, and future planning. WHO Europe is actively working to raise awareness about the negative health and well‐being impacts of prolonged caregiving, aiming to improve access to training and support for informal caregivers, providing the required skills and knowledge to deliver care whilst simultaneously supporting their well‐being and self‐care [53]. WHO Europe is currently seeking to support the informal caregivers through innovative interventions, whilst engaging the informal caregivers in co‐development and co‐production of policies and support materials, to ensure that interventions are not only relevant but also effective [55]. This approach could be vital to ensure the caregivers' need for validation and recognition, as described in the studies (1, 3–6, 8–16). Furthermore, WHO Europe presents a strategy aimed at understanding the caregivers' needs, providing respite care and training, offering counselling and emotional support, ensuring case management, service coordination and information about available services, providing financial support, as well as regular health checkups [53].

WHO Europe's strategy aligns with the findings of this review, which also underscores the multifaceted support needs of spousal caregivers to people with YOD. The focus on personalised support resonates with the need for tailored interventions that address emotional, practical, and informational aspects of caregiving, ultimately aiming to improve caregivers' quality of life and well‐being. This is particularly relevant to informal caregivers of people with YOD, who often face a different set of challenges than those of informal caregivers of older people with dementia. However, the findings of this review highlight a gap between policy goals and the lived experiences of these caregivers, emphasising the need for the development of tailored interventions that span across the multifaceted needs of the informal caregivers to persons with YOD on both personal, social, and systemic levels.

### 4.4 Critical Reflections and Limitations

This scoping review was conducted with pragmatic limitations. Only peer‐reviewed articles in English or Scandinavian languages were eligible, due to the language constraints of the researchers. While the peer reviewing of the included publications supports the quality of the findings, including publications in more languages and additional empirical data from the included publications may have provided further insights into the lived experiences of spousal caregivers. However, transparency and rigour were prioritised at each stage, and efforts were made to reduce bias in the study selection, data extraction, and critical appraisal process by researcher triangulation. Moreover, a structured and rigorous approach was guided by utilising the JBI manual [29].

While the scoping review was conducted with an inductive approach, the researchers' perceptions and experiences inevitably affect the interpretation of data [58]. Hence, this influence was sought to be minimised by opting for transparency and reflexivity throughout the research process.

#### 4.4.1 Quality Assessment

The studies included in this scoping review were primarily qualitative, except for a single cross‐sectional study. The qualitative studies underwent critical appraisal to assess their quality and trustworthiness. A pattern can be observed in Figure 2, showing that most “Can't tell” ratings were given for questions 6 and 7, which assess:

- Has the relationship between the researcher and participants been adequately considered?
- Have ethical issues been taken into consideration?

In qualitative research, the researcher's role and ethical considerations are crucial, as they shape interactions with participants and significantly influence the study's process and outcomes. Addressing these aspects transparently is essential for reflecting on the researcher's impact on the findings. While the “Can't tell” rating does not necessarily imply that the researchers have not taken ethical issues or relationships with the participants into consideration it does highlight that they were not explicitly discussed and presented.

### 4.5 Implications for Future Research

To further expand and deepen the understanding of informal caregivers for people with YOD, future reviews could consider a broader scope. This might include studies focusing on interventions and experiences with such interventions, as well as the experiences of people with YOD and other groups of informal caregivers. Such an expansion would offer a more comprehensive overview of the existing knowledge in this area.

Additionally, the gendered imbalance in caregiving and its impact on the caregiving experience highlight an important area for further investigation. Understanding how caregiving roles and experiences differ across genders can help identify specific support needs, challenges, and barriers to accessing care. This, in turn, can inform the development of more targeted and equitable interventions, ensuring that support services are responsive to the diverse realities of caregivers. By utilising the mapped findings of this scoping review, future research can help develop a more evidence‐based approach to future services and support, in what context they should be provided, and how and when they should be offered.

## 5 Conclusion

This scoping review identified a broad range of unmet needs and challenges faced by spousal caregivers of persons with YOD, highlighting the need for psychoeducational, social, practical, emotional, and navigational support. The scope of identified themes reflects the complex nature of the caregiving experience and the significant disruption that being a spousal caregiver to a person with YOD creates in everyday living. The review demonstrates gaps in current support systems, spanning personal, social, and systemic levels. Addressing these needs is essential to ensure the well‐being of spousal caregivers and to ensure the sustainability and quality of informal care to people with YOD. Based on this review, future interventions should focus on accessible, comprehensive, and tailored support services that address the unique challenges of YOD caregiving.

## Author Contributions

Both authors contributed to the background and aim of the review.

## Disclosure

*Permission to Reproduce Material From Other Sources:* Not relevant in this review.

## Ethics Statement

The authors have nothing to report.

## Consent

The authors have nothing to report.

## Conflicts of Interest

The authors declare no conflicts of interest.

## Supporting information


Appendix S1.

## Data Availability

The data that supports the findings of this study are available in the Supporting Information of this article.

## References

[1] WHO , Global Action Plan on the Public Health Response to Dementia 2017–2025 (2017), https://iris.who.int/bitstream/handle/10665/259615/9789241513487‐eng.pdf?sequence=1.

[2] N. Agrawal and K. Jefferies , “Early‐Onset Dementia,” Advances in Psychiatric Treatment 15, no. 5 (2009): 380–388, 10.1192/apt.bp.107.004572.

[3] R. Koopmans and T. Rosness , “Young Onset Dementia – What Does the Name Imply?,” International Psychogeriatrics 26, no. 12 (2014): 1931–1933, 10.1017/S1041610214001574. 25382199

[4] S. Hendriks , K. Peetoom , C. Bakker , et al., “Global Prevalence of Young‐Onset Dementia,” JAMA Neurology 78, no. 9 (2021): 1080, 10.1001/jamaneurol.2021.2161. 34279544 PMC8290331

[5] K. Burkinshaw , G. Tsourtos , and M. Cations , “System and Policy‐Level Barriers and Facilitators for Timely and Accurate Diagnosis of Young Onset Dementia,” International Journal of Geriatric Psychiatry 38, no. 1 (2023): e5859, 10.1002/gps.5859. 36484460

[6] C. Bakker , R. T. C. M. Koopmans , Y. A. L. Pijnenburg , et al., “Time to Diagnosis in Young‐Onset Dementia as Compared With Late‐Onset Dementia,” Psychological Medicine 43, no. 2 (2013): 423–432, 10.1017/S0033291712001122. 22640548

[7] B. Draper , M. Cations , F. White , et al., “Time to Diagnosis in Young‐Onset Dementia and Its Determinants: The INSPIRED Study,” International Journal of Geriatric Psychiatry 31, no. 11 (2016): 1217–1224, 10.1002/gps.4430. 26807846

[8] B. Draper and A. Withall , “Young Onset Dementia,” Internal Medicine Journal 46, no. 7 (2016): 779–786, 10.1111/imj.13099. 27405890

[9] D. van Vliet , M. E. de Vugt , C. Bakker , et al., “Time to Diagnosis in Young‐Onset Dementia as Compared With Late‐Onset Dementia,” Psychological Medicine 43, no. 2 (2013): 423–432, 10.1017/s0033291712001122. 22640548

[10] C. Kilty , S. Cahill , T. Foley , and S. Fox , “Young Onset Dementia: Implications for Employment and Finances,” Dementia (London) 22, no. 1 (2023): 68–84, 10.1177/14713012221132374. 36254673 PMC9772889

[11] S. Novek and V. H. Menec , “Age, Dementia, and Diagnostic Candidacy: Examining the Diagnosis of Young Onset Dementia Using the Candidacy Framework,” Qualitative Health Research 31, no. 3 (2021): 498–511, 10.1177/1049732320970199. 33213257

[12] V. Bakker , D. Vugt , V. Vliet , et al., “The Use of Formal and Informal Care in Early Onset Dementia: Results From the NeedYD Study,” American Journal of Geriatric Psychiatry 21, no. 1 (2013): 37–45, 10.1016/j.jagp.2012.10.004. 23290201

[13] N. R. Kimura , V. L. Maffioletti , R. L. Santos , M. A. Baptista , and M. C. Dourado , “Psychosocial Impact of Early Onset Dementia Among Caregivers,” Trends in Psychiatry and Psychotherapy 37, no. 4 (2015): 213–219, 10.1590/2237-6089-2015-0038. 26689390

[14] L. Lim , A. Zhang , L. Lim , et al., “High Caregiver Burden in Young Onset Dementia: What Factors Need Attention?,” Journal of Alzheimer's Disease 61, no. 2 (2018): 537–543, 10.3233/jad-170409. 29171995

[15] M. F. Johansson , K. J. McKee , L. Dahlberg , et al., “A Comparison of Spouse and Non‐Spouse Carers of People With Dementia: A Descriptive Analysis of Swedish National Survey Data,” BMC Geriatrics 21, no. 1 (2021): 338, 10.1186/s12877-021-02264-0. 34078292 PMC8170983

[16] D. Gallagher , A. Ni Mhaolain , L. Crosby , et al., “Self‐Efficacy for Managing Dementia May Protect Against Burden and Depression in Alzheimer's Caregivers,” Aging & Mental Health 15, no. 6 (2011): 663–670, 10.1080/13607863.2011.562179. 21547745

[17] S. Kaiser and P. K. Panegyres , “The Psychosocial Impact of Young Onset Dementia on Spouses,” American Journal of Alzheimer's Disease & Other Dementias 21, no. 6 (2007): 398–402, 10.1177/1533317506293259. 17267371

[18] M. L. Barca , K. Thorsen , K. Engedal , P. K. Haugen , and A. Johannessen , “Nobody Asked Me How I Felt: Experiences of Adult Children of Persons With Young‐Onset Dementia,” International Psychogeriatrics 26, no. 12 (2014): 1935–1944, 10.1017/s1041610213002639. 24423756

[19] C. Blake and L. Hopper , “Childhood Perspectives of Parental Young Onset Dementia: A Qualitative Data Synthesis,” Dementia (London) 21, no. 4 (2022): 1304–1327, 10.1177/14713012221077531. 35332810 PMC9109219

[20] H. Groennestad and W. Malmedal , “Having a Parent With Early‐Onset Dementia: A Qualitative Study of Young Adult Children,” Nursing Research and Practice 2022 (2022): 7945773, 10.1155/2022/7945773. 35957655 PMC9357811

[21] Å. Grundberg , J. Sandberg , and G. Craftman Å , “Childrens' and Young Adults' Perspectives of Having a Parent With Dementia Diagnosis: A Scoping Review,” Dementia (London) 20, no. 8 (2021): 2933–2956, 10.1177/14713012211023653. 34096358

[22] P. Sikes and M. Hall , “The Impact of Parental Young Onset Dementia on Children and Young People's Educational Careers,” British Educational Research Journal 44, no. 4 (2018): 593–607, 10.1002/berj.3448. 30147206 PMC6099504

[23] H. J. Aslett , J. C. Huws , R. T. Woods , and J. Kelly‐Rhind , “‘This Is Killing Me Inside’: The Impact of Having a Parent With Young‐Onset Dementia,” Dementia (London) 18, no. 3 (2019): 1089–1107, 10.1177/1471301217702977. 28871812

[24] A. Johannessen , K. Engedal , and K. Thorsen , “Adult Children of Parents With Young‐Onset Dementia Narrate the Experiences of Their Youth Through Metaphors,” Journal of Multidisciplinary Healthcare 8 (2015): 245–254, 10.2147/jmdh.S84069. 26060403 PMC4454217

[25] M. Wiggins , A. McEwen , and A. Sexton , “Young‐Onset Dementia: A Systematic Review of the Psychological and Social Impact on Relatives,” Patient Education and Counseling 107 (2023): 107585, 10.1016/j.pec.2022.107585. 36516659

[26] V. Bakker , D. Vugt , V. Vliet , et al., “Predictors of the Time to Institutionalization in Young‐ Versus Late‐Onset Dementia: Results From the Needs in Young Onset Dementia (NeedYD) Study,” Journal of the American Medical Directors Association 14, no. 4 (2013): 248–253, 10.1016/j.jamda.2012.09.011. 23123009

[27] M. Cations , A. Withall , R. Horsfall , et al., “Why Aren't People With Young Onset Dementia and Their Supporters Using Formal Services? Results From the INSPIRED Study,” PLoS One 12, no. 7 (2017): e0180935, 10.1371/journal.pone.0180935. 28723931 PMC5517136

[28] M. D. J. Peters , C. M. Godfrey , H. Khalil , P. McInerney , D. Parker , and C. B. Soares , “Guidance for Conducting Systematic Scoping Reviews,” JBI Evidence Implementation 13, no. 3 (2015): 141–146, 10.1097/xeb.0000000000000050. 26134548

[29] JBI , JBI Manual for Evidence Synthesis, ed. L. C. Aromataris , K. Porritt , B. Pilla , and Z. Jordan (JBI, 2024), 10.46658/JBIMES-24-01.

[30] A. Ohman , “Qualitative Methodology for Rehabilitation Research,” Journal of Rehabilitation Medicine 37, no. 5 (2005): 273–280, 10.1080/16501970510040056. 16208859

[31] EndNote , “EndNote,” (2013), In (Version EndNote 20) [64 bit]. Clarivate Analytics.

[32] Z. Munn , E. Aromataris , C. Tufanaru , et al., “The Development of Software to Support Multiple Systematic Review Types: The Joanna Briggs Institute System for the Unified Management, Assessment and Review of Information (JBI SUMARI),” International Journal of Evidence‐Based Healthcare 17, no. 1 (2019): 36–43, 10.1097/xeb.0000000000000152. 30239357

[33] M. J. Page , M. J. Page , P. M. Bossuyt , et al., “The PRISMA 2020 Statement: An Updated Guideline for Reporting Systematic Reviews,” BMJ (Clinical Research Ed.) 372 (2021): n71. 10.1136/bmj.n71 PMC8005924 33782057

[34] CASP , CASP Qualitative Studies Checklist (2023), https://casp‐uk.net/casp‐tools‐checklists/qualitative‐studies‐checklist/.

[35] S. Moola , Z. Munn , C. Tufanaru , et al., “Chapter 7: Systematic Reviews of Etiology and Risk,” in JBI Manual for Evidence Synthesis, ed. E. Aromataris , C. Lockwood , K. Porritt , B. Pilla , and Z. Jordan (JBI, 2020), https://synthesismanual.jbi.global.

[36] S. Elo and H. Kyngäs , “The Qualitative Content Analysis Process,” Journal of Advanced Nursing 62, no. 1 (2008): 107–115, 10.1111/j.1365-2648.2007.04569.x. 18352969

[37] M. Aspö , L. N. C. Visser , M. Kivipelto , A. M. Boström , and B. Seiger Cronfalk , “Family Members' Experiences of Young‐Onset Dementia: Becoming Responsible Yet Feeling Powerless,” Journal of Multidisciplinary Healthcare 16 (2023): 2379–2390, 10.2147/jmdh.S418285. 37609051 PMC10441645

[38] M. Bayly , M. E. O'Connell , A. Kortzman , S. Peacock , D. G. Morgan , and A. Kirk , “Family Carers' Narratives of the Financial Consequences of Young Onset Dementia,” Dementia (London) 20, no. 8 (2021): 2708–2724, 10.1177/14713012211009341. 33877946 PMC8670747

[39] I. Chirico , G. Ottoboni , S. Linarello , E. Ferriani , E. Marrocco , and R. Chattat , “Family Experience of Young‐Onset Dementia: The Perspectives of Spouses and Children,” Aging & Mental Health 26, no. 11 (2022): 2243–2251, 10.1080/13607863.2021.2008871. 34842004

[40] F. Ducharme , M. J. Kergoat , P. Antoine , F. Pasquier , and R. Coulombe , “The Unique Experience of Spouses in Early‐Onset Dementia,” American Journal of Alzheimer's Disease and Other Dementias 28, no. 6 (2013): 634–641, 10.1177/1533317513494443. PMC10852552 23823140

[41] F. Ducharme , M. J. Kergoat , R. Coulombe , L. Lévesque , P. Antoine , and F. Pasquier , “Unmet Support Needs of Early‐Onset Dementia Family Caregivers: A Mixed‐Design Study,” BMC Nursing 13, no. 1 (2014): 49, 10.1186/s12912-014-0049-3. 25550685 PMC4279790

[42] R. Flynn and H. Mulcahy , “Early‐Onset Dementia: The Impact on Family Care‐Givers,” British Journal of Community Nursing 18, no. 12 (2013): 598–606, 10.12968/bjcn.2013.18.12.598. 24335793

[43] A. K. Gibson , K. A. Anderson , and S. Acocks , “Exploring the Service and Support Needs of Families With Early‐Onset Alzheimer's Disease,” American Journal of Alzheimer's Disease and Other Dementias 29, no. 7 (2014): 596–600, 10.1177/1533317514558160. PMC10852964 25392308

[44] V. A. Grunberg , S. M. Bannon , M. Reichman , P. J. Popok , and A. M. Vranceanu , “Psychosocial Treatment Preferences of Persons Living With Young‐Onset Dementia and Their Partners,” Dementia (London) 21, no. 1 (2022): 41–60, 10.1177/14713012211027007. 34151598 PMC10289008

[45] A. Johannessen , A. S. Helvik , K. Engedal , and K. Thorsen , “Experiences and Needs of Spouses of Persons With Young‐Onset Frontotemporal Lobe Dementia During the Progression of the Disease,” Scandinavian Journal of Caring Sciences 31, no. 4 (2017): 779–788, 10.1111/scs.12397. 28276143

[46] C. Kilty , P. Boland , J. Goodwin , and Á. de Róiste , “Caring for People With Young Onset Dementia: An Interpretative Phenomenological Analysis of Family Caregivers' Experiences,” Journal of Psychosocial Nursing and Mental Health Services 57, no. 11 (2019): 37–44, 10.3928/02793695-20190821-02. 31437284

[47] S. Lockeridge and J. Simpson , “The Experience of Caring for a Partner With Young Onset Dementia: How Younger Carers Cope,” Dementia (London) 12, no. 5 (2013): 635–651, 10.1177/1471301212440873. 24337334

[48] S. Novek and V. Menec , “Conceptualizing Access to Community‐Based Supports From the Perspectives of People Living With Young Onset Dementia, Family Members and Providers,” Dementia (London) 22, no. 1 (2023): 180–196, 10.1177/14713012221138145. 36377262 PMC9772890

[49] P. J. Popok , M. Reichman , L. LeFeber , V. A. Grunberg , S. M. Bannon , and A. M. Vranceanu , “One Diagnosis, Two Perspectives: Lived Experiences of Persons With Young‐Onset Dementia and Their Care‐Partners,” Gerontologist 62, no. 9 (2022): 1311–1323, 10.1093/geront/gnac050. 35442443 PMC9579459

[50] K. Thorsen and A. Johannessen , “How Gender Matters in Demanding Caring for a Spouse With Young‐Onset Dementia. A Narrative Study,” Journal of Women & Aging 35, no. 1 (2023): 81–97, 10.1080/08952841.2022.2087455. 35722752

[51] E. Wawrziczny , F. Pasquier , F. Ducharme , M. J. Kergoat , and P. Antoine , “From ‘Needing to Know’ to ‘Needing Not to Know More’: An Interpretative Phenomenological Analysis of Couples' Experiences With Early‐Onset Alzheimer's Disease,” Scandinavian Journal of Caring Sciences 30, no. 4 (2016): 695–703, 10.1111/scs.12290. 26453315

[52] M. Bury , “Chronic Illness as Biographical Disruption,” Sociology of Health & Illness 4, no. 2 (1982): 167–182, 10.1111/1467-9566.ep11339939. 10260456

[53] WHO , Caregiving Impacts on Unpaid Informal Carers' Health and Well‐Being – A Gender Perspective: Factsheet, ed. W. H. O. E. Region (2022).

[54] L. M. Peña‐Longobardo and J. Oliva‐Moreno , “The Economic Value of Non‐Professional Care: A Europe‐Wide Analysis,” International Journal of Health Policy and Management 11, no. 10 (2022): 2272–2286, 10.34172/ijhpm.2021.149. 34814681 PMC9808255

[55] WHO , What It Is Like to Be a Spousal Carer – Perspectives From 2 Women From Ireland and The United Kingdom of Great Britain and Northern Ireland (2023), https://www.who.int/europe/news‐room/feature‐stories/item/what‐it‐is‐like‐to‐be‐a‐spousal‐carer‐‐‐perspectives‐from‐2‐women‐from‐ireland‐and‐the‐united‐kingdom‐of‐great‐britain‐and‐northern‐ireland.

[56] E. Wawrziczny , G. Berna , F. Ducharme , M. J. Kergoat , F. Pasquier , and P. Antoine , “Modeling the Distress of Spousal Caregivers of People With Dementia,” Journal of Alzheimer's Disease 55, no. 2 (2017): 703–716, 10.3233/jad-160558. 27716667

[57] WHO , Shining Light on Women's Contributions: Celebrating Their Role in Informal Care (2024), https://www.who.int/europe/news/item/08‐03‐2024‐shining‐light‐on‐women‐s‐contributions‐‐celebrating‐their‐role‐in‐informal‐care.

[58] R. Berger , “Now I See It, Now I Don't: Researcher's Position and Reflexivity in Qualitative Research,” Qualitative Research 15 (2013): 219–234, 10.1177/1468794112468475.

