# Supplementary material for: Navigating Everyday as Partners to Persons With Early Dementia: A Scoping Review
Source: Scand J Caring Sci. 2025 Aug 5;39(3):e70089. doi: 10.1111/scs.70089 (PMC12325816; doi:10.1111/scs.70089)
Supplement: Supplementary file 1 — Appendix S1. [file SCS-39-0-s001.docx]

# Appendix A – Search Strategies

| **BLOC 1** | **BLOC 2** | **BLOC 3** | **BLOC 4** |
| --- | --- | --- | --- |
| **Dementia** | **Informal caregiver** | **Experience** | **Intervention** |
| ”Early onset dementia” | Spouse | Quality of life | Support |
| ”Early onset Alzheimer” | Spousal | Psychosocial | Support-system |
| ”Young onset dementia” | Husband | Lived experience | Program |
| ”Young onset Alzheimer” | Wife | Well-being | Intervention |
|  | Married | Emotion* | Training |
|  | Partner | Coping | Skills |
|  | Care-partner | Needs | Strategies |
|  | Family |  |  |
|  | Loved ones |  |  |
|  | Next-of-kin |  |  |
|  | Relatives |  |  |
|  | Informal caregivers |  |  |

|  | [Pubmed 01.03.24] **Bloc 1** | | |
| --- | --- | --- | --- |
| **#** | **Search terms** | **Hits** | **Comments** |
| 1 | “Early onset dementia” | 676 |  |
| 2 | “Early onset Alzheimer” | 1201 |  |
| 3 | “Young onset Dementia” | 389 |  |
| 4 | “Young onset alzheimer” | 54 |  |
| 5 | ((("early onset dementia") OR ("early onset alzheimer")) OR ("young onset dementia")) OR ("young onset alzheimer") | 2165 | **BLOC 1** |

|  | [Pubmed 01.03.24] **BLOC 2** | | |
| --- | --- | --- | --- |
| **#** | **Search terms** | **Hits** | **Comments** |
| 1 | Spouse | 37890 | MeSH: Spouses |
| 2 | Spousal | 4485 | Søger kun "spousal"[All Fields] |
| 3 | Husband | 33320 | MeSH: Spouses |
| 4 | Wife | 26824 | MeSH: Spouses |
| 5 | Married | 70116 | MeSH: Marriage |
| 6 | Partner | 259999 |  |
| 7 | Care-partner | 488 | Only "care-partner"[All Fields] |
| 8 | Family | 1838263 | MeSH: Family |
| 9 | Loved ones | 3304 | MeSH: love |
| 10 | Next-of-kin | 2145 | Only "next-of-kin"[All Fields] |
| 11 | Relatives | 3181991 | MeSH: Family |
| 12 | Informal caregivers | 99812 | MeSH: Caregivers |
| 14 | **(((((((((((spouse) OR (spousal)) OR (husband)) OR (wife)) OR (married)) OR (partner)) OR (care-partner)) OR (family)) OR (loved ones)) OR (next-of-kin)) OR (relatives)) OR (informal caregivers)** | **3708230** | **BLOC 2** |

|  | **[Pubmed 01.03.24] BLOC 3** | | |
| --- | --- | --- | --- |
| **#** | **Search terms** | **Hits** | **Comments** |
| 1 | Quality of life | 573990 |  |
| 2 | Psychosocial | 142953 |  |
| 3 | Lived experience | 75540 |  |
| 4 | Well-being | 9615685 | Well-being, health, well, well being |
| 5 | Emotion* | 311794 |  |
| 6 | Support | 11815200 |  |
| 7 | Coping | 78966 | MeSH: Coping skills |
| 8 | Needs | 1334430 |  |
| 9 | **( ((((((quality of life) OR (psychosocial)) OR (lived experience)) OR (well-being)) OR (emotion)) OR (Coping)) OR (needs) )** | **10736758** | **BLOK 3** |

|  | [Pubmed 14.03.2024] – **Bloc 4** | | |
| --- | --- | --- | --- |
| **#** | **Search terms** | **Hits** | **Comments** |
| 1 | Support | 11825305 |  |
| 2 | Program | 1967443 |  |
| 3 | Intervention | 10724999 |  |
| 4 | Training | 2680917 |  |
| 5 | Skills | 311264 |  |
| 6 | Strategies | 1589396 |  |
| **7** | **(((((support) OR (program)) OR (intervention)) OR (training)) OR (skills)) OR (strategies)** | 20293635 | **BLOC 4** |

|  | **[Pubmed 15.03.2024] Final** | | |
| --- | --- | --- | --- |
| **#** | **Search terms** | **Hits** | **Comments** |
| 1 | ((("early onset dementia") OR ("early onset alzheimer")) OR ("young onset dementia")) OR ("young onset alzheimer") | 2171 | BLOC 1 |
| 2 | (((((((((((spouse) OR (spousal)) OR (husband)) OR (wife)) OR (married)) OR (partner)) OR (care-partner)) OR (family)) OR (loved ones)) OR (next-of-kin)) OR (relatives)) OR (informal caregivers) | 3708230 | BLOC 2 |
|  | ( ((((((quality of life) OR (psychosocial)) OR (lived experience)) OR (well-being)) OR (emotion*)) OR (Coping)) OR (needs) ) | 10602920 | BLOC 3 |
| 3 | ((((((support) OR (support-system)) OR (program)) OR (intervention)) OR (training)) OR (skills)) OR (strategies) | 20295153 | BLOC 4 |
| 4 | (((((("early onset dementia") OR ("early onset alzheimer")) OR ("young onset dementia")) OR ("young onset alzheimer")) AND ((((((((((((spouse) OR (spousal)) OR (husband)) OR (wife)) OR (married)) OR (partner)) OR (care-partner)) OR (family)) OR (loved ones)) OR (next-of-kin)) OR (relatives)) OR (informal caregivers))) AND (( ((((((quality of life) OR (psychosocial)) OR (lived experience)) OR (well-being)) OR (emotion*)) OR (Coping)) OR (needs) ))) AND (((((((support) OR (support-system)) OR (program)) OR (intervention)) OR (training)) OR (skills)) OR (strategies)) | 489 | Al blocs |
| 5 | **(((((("early onset dementia") OR ("early onset alzheimer")) OR ("young onset dementia")) OR ("young onset alzheimer")) AND ((((((((((((spouse) OR (spousal)) OR (husband)) OR (wife)) OR (married)) OR (partner)) OR (care-partner)) OR (family)) OR (loved ones)) OR (next-of-kin)) OR (relatives)) OR (informal caregivers))) AND (( ((((((quality of life) OR (psychosocial)) OR (lived experience)) OR (well-being)) OR (emotion*)) OR (Coping)) OR (needs) ))) AND (((((((support) OR (support-system)) OR (program)) OR (intervention)) OR (training)) OR (skills)) OR (strategies))** | 366 | Al blocs  Filtre years: 2013-2023 |

|  | **[Academic Search Premier, APA PsycInfo, CINAHL Ultimate, MEDLINE via EBSCOhost 15.03.2024]** | | |
| --- | --- | --- | --- |
| **#** | **Search terms** | **Hits** | **Comments** |
| 1 | **(((((("early onset dementia") OR ("early onset alzheimer")) OR ("young onset dementia")) OR ("young onset alzheimer")) AND ((((((((((((spouse) OR (spousal)) OR (husband)) OR (wife)) OR (married)) OR (partner)) OR (care-partner)) OR (family)) OR (loved ones)) OR (next-of-kin)) OR (relatives)) OR (informal caregivers))) AND (( ((((((quality of life) OR (psychosocial)) OR (lived experience)) OR (well-being)) OR (emotion*)) OR (Coping)) OR (needs) ))) AND (((((((support) OR (support-system)) OR (program)) OR (intervention)) OR (training)) OR (skills)) OR (strategies))** | 431 |  |
| 2 | **(((((("early onset dementia") OR ("early onset alzheimer")) OR ("young onset dementia")) OR ("young onset alzheimer")) AND ((((((((((((spouse) OR (spousal)) OR (husband)) OR (wife)) OR (married)) OR (partner)) OR (care-partner)) OR (family)) OR (loved ones)) OR (next-of-kin)) OR (relatives)) OR (informal caregivers))) AND (( ((((((quality of life) OR (psychosocial)) OR (lived experience)) OR (well-being)) OR (emotion*)) OR (Coping)) OR (needs) ))) AND (((((((support) OR (support-system)) OR (program)) OR (intervention)) OR (training)) OR (skills)) OR (strategies))** | 326 | Filtre years: 2013-2023 |

|  | **[Embase 15.03.2024]** | | |
| --- | --- | --- | --- |
| **#** | **Search terms** | **Hits** | **Comments** |
| 1 | **(((((("early onset dementia") OR ("early onset alzheimer")) OR ("young onset dementia")) OR ("young onset alzheimer")) AND ((((((((((((spouse) OR (spousal)) OR (husband)) OR (wife)) OR (married)) OR (partner)) OR (care-partner)) OR (family)) OR (loved ones)) OR (next-of-kin)) OR (relatives)) OR (informal caregivers))) AND (( ((((((quality of life) OR (psychosocial)) OR (lived experience)) OR (well-being)) OR (emotion*)) OR (Coping)) OR (needs) ))) AND (((((((support) OR (support-system)) OR (program)) OR (intervention)) OR (training)) OR (skills)) OR (strategies))** | 141 |  |
| 2 | **(((((("early onset dementia") OR ("early onset alzheimer")) OR ("young onset dementia")) OR ("young onset alzheimer")) AND ((((((((((((spouse) OR (spousal)) OR (husband)) OR (wife)) OR (married)) OR (partner)) OR (care-partner)) OR (family)) OR (loved ones)) OR (next-of-kin)) OR (relatives)) OR (informal caregivers))) AND (( ((((((quality of life) OR (psychosocial)) OR (lived experience)) OR (well-being)) OR (emotion*)) OR (Coping)) OR (needs) ))) AND (((((((support) OR (support-system)) OR (program)) OR (intervention)) OR (training)) OR (skills)) OR (strategies))** | 118 | Filtre years: 2013-2023 |

|  | **[Scopus 15.03.2024]** | | |
| --- | --- | --- | --- |
| **#** | **Search terms** | **Hits** | **Comments** |
| 1 | **(((((("early onset dementia") OR ("early onset alzheimer")) OR ("young onset dementia")) OR ("young onset alzheimer")) AND ((((((((((((spouse) OR (spousal)) OR (husband)) OR (wife)) OR (married)) OR (partner)) OR (care-partner)) OR (family)) OR (loved ones)) OR (next-of-kin)) OR (relatives)) OR (informal caregivers))) AND (( ((((((quality of life) OR (psychosocial)) OR (lived experience)) OR (well-being)) OR (emotion*)) OR (Coping)) OR (needs) ))) AND (((((((support) OR (support-system)) OR (program)) OR (intervention)) OR (training)) OR (skills)) OR (strategies))** | 152 |  |
| 2 | **(((((("early onset dementia") OR ("early onset alzheimer")) OR ("young onset dementia")) OR ("young onset alzheimer")) AND ((((((((((((spouse) OR (spousal)) OR (husband)) OR (wife)) OR (married)) OR (partner)) OR (care-partner)) OR (family)) OR (loved ones)) OR (next-of-kin)) OR (relatives)) OR (informal caregivers))) AND (( ((((((quality of life) OR (psychosocial)) OR (lived experience)) OR (well-being)) OR (emotion*)) OR (Coping)) OR (needs) ))) AND (((((((support) OR (support-system)) OR (program)) OR (intervention)) OR (training)) OR (skills)) OR (strategies))** | 119 | Filtre years: 2013-2023 |

# Appendix B – Critical appraisal

**CASP QUALITATIVE CHECKLIST**

| Author | Q1 | Q2 | Q3 | Q4 | Q5 | Q6 | Q7 | Q8 | Q9 | Q10 | Assessment |
| --- | --- | --- | --- | --- | --- | --- | --- | --- | --- | --- | --- |
| Aspö, M. et al., 2023 | Y | Y | Y | Y | Y | U | Y | Y | Y | Y |  |
| Bayly, M. et al, 2021 | Y | Y | Y | Y | U | U | U | Y | Y | Y |  |
| Chirico, I. et al, 2022 | Y | Y | Y | Y | Y | U | Y | Y | Y | Y |  |
| Ducharme, F. et al, 2013 | Y | Y | Y | Y | Y | Y | U | Y | Y | Y |  |
| Ducharme, F. et al, 2014 | Y | Y | Y | Y | Y | U | Y | Y | Y | Y |  |
| Flynn & Mulcahy, 2013 | Y | Y | Y | Y | Y | U | Y | Y | Y | Y |  |
| Gibson, A. et al, 2014 |  |  |  |  |  |  |  |  |  |  | OBS cross-sectional |
| Grunberg, V. et al, 2022 | Y | Y | Y | Y | Y | Y | U | Y | Y | Y |  |
| Johannesen, A. et al, 2017 | Y | Y | Y | Y | Y | Y | Y | Y | Y | Y |  |
| Kilty, C. et al, 2019 | Y | Y | Y | Y | Y | U | Y | Y | Y | Y |  |
| Kimura, N. et al, 2019 | Y | Y | Y | U | Y | Y | Y | Y | Y | Y |  |
| Lockeridge, S. & Simpson, J., 2013 | Y | Y | Y | Y | Y | Y | U | Y | U | Y |  |
| Novek, S. & Menec, V., 2022 | Y | Y | Y | Y | Y | U | Y | Y | Y | Y |  |
| Popok, PJ et al., 2022 | Y | Y | Y | Y | Y | Y | U | Y | Y | Y |  |
| Thorsen, K & Johannessen, A, 2023 | Y | Y | Y | U | Y | U | Y | Y | U | U |  |
| A Wawrziczny, E. et al, 2016 | Y | Y | Y | Y | Y | Y | Y | Y | Y | Y |  |
| B Wawrziczny, E. et al, 2016 | Y | Y | Y | Y | Y | U | Y | Y | Y | U |  |

Critical Appraisal Skills Programme (2018). CASP (Qualitative) Checklist. [online] Available at: URL. Accessed: <https://casp-uk.net/checklists/casp-qualitative-studies-checklist-fillable.pdf> Date Accessed. 21/08-2024

1. Was there a clear statement of the aims of the research?
2. Is a qualitative methodology appropriate?
3. Was the research design appropriate to address the aims of the research?
4. Was the recruitment strategy appropriate to the aims of the research?
5. Was the data collected in a way that addressed the research issue?
6. Has the relationship between researcher and participants been adequately considered?
7. Have ethical issues been taken into consideration?
8. Was the data analysis sufficiently rigorous?
9. Is there a clear statement of findings?
10. How valuable is the research?

(**Y** Yes, **N** No, **U** Unclear)

**JBI Checklist FOR ANALYTICAL CROSS-SECTIONAL STUDIES**

| Author | Q1 | Q2 | Q3 | Q4 | Q5 | Q6 | Q7 | Q8 | Assessment |
| --- | --- | --- | --- | --- | --- | --- | --- | --- | --- |
| Gibson, A. et al, 2014 | U | Y | NA | Y | Y | N | Y | Y | Include |

Moola S, Munn Z, Tufanaru C, Aromataris E, Sears K, Sfetcu R, Currie M, Qureshi R, Mattis P, Lisy K, Mu P-F. Chapter 7: Systematic reviews of etiology and risk. In: Aromataris E, Munn Z (Editors). JBI Manual for Evidence Synthesis. JBI, 2020. Available from <https://synthesismanual.jbi.global> Date Accessed. 02/09-2024

1. Were the criteria for inclusion in the sample clearly defined?

2. Were the study subjects and the setting described in detail?

3. Was the exposure measured in a valid and reliable way?

4. Were objective, standard criteria used for measurement of the condition?

5. Were confounding factors identified?

6. Were strategies to deal with confounding factors stated?

7. Were the outcomes measured in a valid and reliable way?

8. Was appropriate statistical analysis used?

(**Y** Yes, **N** No, **U** Unclear, **NA** Not applicable)
